# Predicting Measles Vaccination Defaulting in Uganda and Kenya: Comparative Evaluation of Custom Soft Voting and TabNet Stacked Ensemble Architectures

**DOI:** 10.2196/95727

**Published:** 2026-10-13

**Authors:** Bartha Alexandra Nantongo, Josephine Nabukenya, Peter Nabende

**Affiliations:** 1 Department of Computer Science, School of Computing and Information Science, Kyambogo University, P.O Box 1, Kyambogo, Kampala, Uganda, 256 788746136; 2 Department of Information Systems, College of Computing and Information Sciences, Makerere University, Kampala, Kampala, Uganda

**Keywords:** Advanced-ensemble-learning, TabNet, deep neural networks, measles, vaccination-defaulting, Tabular Network

## Abstract

**Background:**

Measles is a major cause of death among infants despite free routine immunization (RI) in low- and middle-income countries (LMICs). Vaccination defaulting contributes to measles outbreak occurrences. Conventional immunization defaulter identification methods are expensive to implement, prone to data falsification, reactive, and overestimate uptake. Several machine learning studies leveraged the use of child and maternal characteristics as predictors of immunization defaulting, overlooking community, demographic, and socioeconomic factors. Similarly, there is limited research regarding the use of interpretable deep learning and advanced ensemble methods to improve model performance in predicting RI defaulters and establishing significant predictors at the national level in the LMIC context, like Uganda.

**Objective:**

This study predicts infants at a risk of defaulting on measles vaccination in Uganda and Kenya using advanced ensemble learning architectures.

**Methods:**

The study benchmarked classical and deep learning models using Demographic and Health Survey (DHS) datasets (2016-2022) with child, maternal, community, social-economic, and demographic characteristics as predictors. The robust modeling pipeline began with feature selection according to the literature and the expert domain. It was followed by vaccination eligibility, missing value handling, and feature elimination using the phi-k correlation framework. To mitigate class imbalance, synthetic minority over-sampling technique for nominal continuous features was used in a training pipeline after the RobustScaler. Nine baseline classifiers, including Tabular Network (TabNet), multilayer perceptron (MLP), random forest (RF), gradient boosting (GB), adaptive-boosting (AB), among others, underwent stratified k-fold cross-validation and hyperparameter optimization via grid search. Custom soft voting was evaluated alongside TabNet stack architecture for superior ensemble prediction. Models were evaluated for accuracy, precision, recall, and *F*_1_-score. Model explainability was decoded using Shapley additive explanations (SHAP), while Streamlit and SQLite formed the tool architecture.

**Results:**

The proposed TabNet+GB+RF custom soft voting achieved significant, nonrandom performance over tuned base models with an *F*_1_-score of 0.7111, area under the curve (AUC) of 0.8847 for Kenya. Conversely, for Uganda, the TabNet hybrid ensemble (GB+RF+ AB→TabNet) was the best with AUC of 0.8862 and *F*_1_-score of 0.6756, which was statistically insignificant. SHAP identified child’s age and vitamin A1 as universal key predictors in both countries; others varied. In Kenya, time to water source, respondents’ education, district lived previously, and current district were the most influential global features, respectively. In Uganda, Polio inactive, Pentavalent3, current district, and respondents’ education were the most influential factors to defaulting, respectively.

**Conclusions:**

The proposed advanced ensemble learning models improved the classification of measles vaccine defaulters compared to individual models. The study recommends distinct country-tailored strategies. In Kenya, outreaches are recommended to areas with low literacy, distant water sources, and mobile populations. Conversely, in Uganda, the study recommends reminders to avoid missing preceding vaccines and outreaches to high-default districts.

## Introduction

### Background

Childhood mortality rates have declined globally. However, the risk of a child dying before the age of one year is highest in Africa at 51 per 1000 live births compared to 8 per 1000 births in the European region [1]. Currently, the probability of a child dying between birth and 5 years in Uganda is 53 per 1000 live births [2], while Kenya reported 41 deaths per 1000 live births [3]. Vaccine-preventable diseases (VPDs) account for 29% of deaths among children aged 1‐59 months and an estimated 3 million deaths every year, mainly in Africa and Asia [4].

Measles vaccination marks a great milestone as it is the last administered among the basic antigens [3] (see Table S1 in Multimedia Appendix 1). Measles is an acute viral VPD whose only reservoir is humans [5]. It is transmitted through the air or direct contact with an infected person [5]. With an incubation period of 7‐21 days [5], the World Health Organization indicates that up to 3 million measles cases are recorded annually [6]. Measles is one of the main causes of death among infants in the prevaccine period due to complications from the respiratory tract (pneumonia), the central nervous system (meningitis, encephalitis, meningoencephalitis, and encephalomyelitis), and the gastrointestinal tract (diarrhea) [6]. Despite free routine immunization (RI), many infants are zero-dose or drop out of the immunization schedule [7]. This situation was compounded by the COVID-19 pandemic, which impaired RI services in African countries, in that only 3 countries met the target of 95% first measles vaccination coverage in 2020 [5]. From January to March 2022, about 17,500 cases of measles were recorded in Africa, while 20 African countries reported measles outbreaks in the first quarter of 2022 [5].

Uganda experienced measles outbreaks in 76 districts in 2018 [8], January 2020 in Nakaseke [5], and February-June 2023 in Bundibugyo [9], among others. These outbreaks reflect the existence of communities with partially vaccinated or zero-dose infants whose herd immunity is not high enough to stall disease transmission [10]. Similarly, in Kenya, 2% of children aged 12‐23 months received no vaccinations [3].

Real-time monitoring of RI enables identification of underserved communities; zero-dose infants are likely to drop out of the immunization schedule [11]. Conventional defaulter identification methods, like resource-based approach (RBA) [12], rapid coverage monitoring (RCM) [13], administrative estimates [11], and surveys, are expensive to implement regularly [11,14], propagate data falsification [15], overestimate uptake [11,12,14], and are not proactive.

Several studies [16-18] have used individual models to predict RI defaulters, predominantly leveraging child and maternal characteristics as predictors, yet overlooking community, socioeconomic, and demographic factors. Some never applied resampling techniques like cross-validation to reduce overfitting or data balancing during training of an imbalanced dataset distribution. In addition, the studies lacked the application of interpretable deep learning methods, significant feature quantifying, and recommending remedial intervention strategies at a national level in Uganda and Kenya concerning measles vaccination defaulting. We claim that using advanced ensemble learning to predict immunization defaulters may reduce the risk of outbreaks and infant mortality.

Nantongo et al established that immunization defaulting increases as the child’s age increases and worsens with measles [19]. The study also indicated the reception of preceding vaccines among predictors for measles in Uganda. However, the model performance for measles was the worst among the four antigens. Nantongo et al [19] did not explore an interpretable deep neural network approach and an advanced ensemble technique; yet, interpretability was key in public health. Neither did it establish if the same predictors would influence measles defaulting in another geographical area other than Uganda, for the models’ generalizability. Accordingly, this study leverages literature to (1) increase predictors’ diversity, (2) explore a comparative study between Uganda and Kenya, (3) experiment with custom soft voting and stacking architectures, (4) experiment performance using interpretable deep learning methods, specifically TabNet, and (5) evaluate performance using a confusion matrix, Shapley additive explanations (SHAP), in addition to other metrics to improve interpretability, to guide public health officers in both countries to mitigate measles defaulting.

### Machine Learning Models for Tabular Data

Traditional machine learning models for tabular data, like tree-based ensembles such as random forest (RF) and gradient boosting (GB), among others, are considered the top-performing models [20]. However, deep learning models like multilayer perceptron (MLP) and Tabular Network (TabNet) have been presented to perform better. MLP is a general-purpose “black-box” neural network characterized by multiple layers where every neuron in one layer is connected to every neuron in the next layer, and is applicable to many data types, but traditionally structured for tabular data due to its simple linear flow.

TabNet is a high-performance interpretable deep neural network for tabular data with feature selection capabilities [20]. Unlike traditional neural networks that process features all at once, TabNet has a sequential attention mechanism that learns to select a subset of the most relevant features to reason from at every decision step, providing local and global interpretability [20] (Figure 1). The TabNet architecture consists of an encoder that consists of a feature transformer, an attentive transformer, and feature masking, and a decoder for self-supervised learning [20]. TabNet has illustrated superior performance over traditional machine learning models [20,21], hence its application in this study.

**Figure 1.**
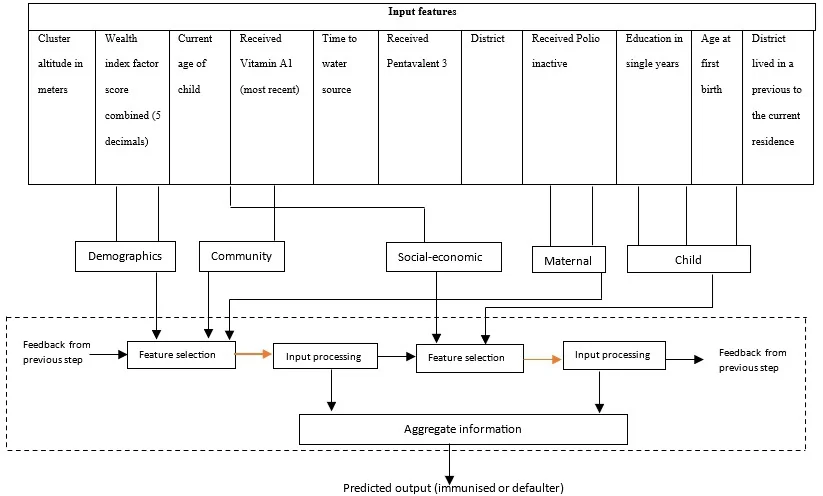
Customized TabNet’s sparse feature selection.

### Related Literature

In the medical field, a comparative performance assessment of a multilevel ensemble learning model to detect diabetes by [22] achieved a maximum accuracy of 88.3% compared to individual models experimented with, and the study suggested the use of synthetic minority over-sampling technique (SMOTE) on imbalanced classification datasets for future work [22]. Similarly, a study [23] concluded that the ensemble learning method was more suitable for handling the classification problems in the bioinformatics domain since it gave better accuracy with a lower root-mean square error.

Correspondingly, another study [24] experimented with stacking linear models, support vector regression (SVR) with a linear kernel, and SVR with a Gaussian kernel. Their results revealed that stack ensemble learning effectively predicted vaccination uptake using web search queries and clinical data for Denmark with a 4.7 root-mean-squared error. In addition, a study forecasting monkeypox transmission in different continents proposed stacking ensemble learning of adaptive boosting (AB) regression, GB regression, RF regression, ordinary least square regression, least absolute shrinkage selection operator regression, and ridge regression. Experimental results revealed that the stacking ensemble learning outperformed other ML approaches [25].

The study by Nantongo et al [19] used demographic, socioeconomic, maternal, community, and child characteristics as predictors. It also experimented with a model with cross-validation and balanced distribution, indicating RF as the best model among the 9 at 84% accuracy in predicting measles vaccination defaulting [19]. As mentioned in the preceding section, there is limited research regarding comparing Uganda and Kenya with reference to measles vaccination defaulting, use of interpretable ensemble deep learning methods to improve model performance with predictor diversity, quantifying significant measles vaccine default predictors with a clear explanation of feature influence on the model outcome, and remedial intervention strategies for policymakers in Uganda and Kenya contexts at a national level. Accordingly, this study aims to predict measles vaccine defaulters in Uganda and Kenya using advanced ensemble learning methods with interpretable influential predictors and context-specific remedial intervention strategies.

### Machine Learning Ensembles

Ensemble methods achieve high accuracy [26]; yet, they require little preprocessing [27]. These methods merge base models’ individual predictions to output a single one using several techniques [28]. The techniques include voting classifiers, bagging and pasting, RFs, boosting, and stacking ensembles. Voting and stacking are nongenerative, while boosting and bagging are generative [28]. The voting classifier employs diverse algorithms trained in parallel and uses either hard or soft voting [27]. Another approach is using the same training algorithm for every prediction, but training them on different random subsets of the training set. Sampling may be done with replacement (bagging) or without (pasting). The RF is an ensemble of decision tree classifiers that uses bagging. Boosting, on the other hand, combines several weak learners into a stronger one trained sequentially, each trying to correct its predecessor [27,29,30].

Stacking is another ensemble method that trains a model to perform prediction aggregation from different base models as inputs for the final predictor (meta learner) [27]. This study experimented with TabNet stack and custom soft voting architectures to predict measles vaccination defaulters in Uganda and Kenya, interpreting influential predictors and suggesting country-tailored remedial intervention strategies for mitigating measles defaulting.

## Methods

### Data Collection and Sampling

Study experiments were conducted using the data collected nationally from the Demographic and Health Survey (DHS) 2022 for Kenya and 2016 for Uganda. DHS data collection uses standardized questionnaires and modules. The study was a retrospective cohort study of infants (living) that were born between 2020‐2022 and 2013‐2016 for Kenya and Uganda, respectively. Although there is a 6-year difference in 2016 Uganda DHS (UDHS) and 2022 Kenya DHS (KDHS), currently the 10% of the 2022 UDHS dataset available [31] for public use may not provide representative estimates at the national level. Experiments under the same conditions were run separately on the country-specific datasets. The study considered immunization uptake of the measles first vaccination administered at 9 months, which marks a great milestone of completion of the basic childhood RI schedule for infants under one year, as indicated in Table S1 in Multimedia Appendix 1.

The 7th KDHS was conducted by the Kenya National Bureau of Statistics (KNBS) in 2022 [3], while the 6th UDHS was conducted by the Uganda Bureau of Statistics (UBOS) in collaboration with the Ministry of Health from June 15 to December 18, 2016 [32]. KDHS 2022 and UDHS 2016 were the most recent national representative datasets at the time of this study’s experiments. The DHS provides a comprehensive overview of population, maternal, and child health issues, and the data are accessible on request to all stakeholders for policy formulation, planning, and measuring progress [32]. The KDHS 2022 collected new indicators not collected in earlier KDHS surveys, like early childhood development, health expenditures, and information on chronic diseases [3], but it maintained the standard indicators for childhood immunization, enabling the current comparative study. Among the many survey objectives, DHS obtains data on many key aspects, like maternal and child health, including immunization coverage among children [32]. The sampling frame used for the 2016 UDHS was the frame of the Uganda National Population and Housing Census (NPHC), conducted in 2014. The census frame was a complete list of all census enumeration areas (EAs) or stratas created for the 2014 NPHC [32]. In Uganda, an EA is a geographic area that covers an average of 130 households [32]. The sample design for the 2022 KDHS provided estimates at the national level, for both urban and rural areas in 47 counties [3]. For purposes of comparative analysis in this study, a county in Kenya corresponds to a district in Uganda. Figure 2 illustrates the architecture of the proposed ensemble.

**Figure 2.**
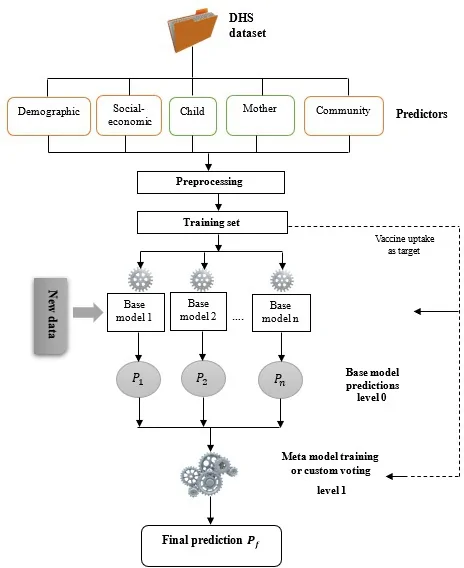
Pipeline for proposed advanced ensemble learning. DHS: Demographic and Health Survey.

### Dataset and Preprocessing

#### Feature Selection

This study selected features predicting vaccine uptake according to the literature [3,7,19,32-37]. Twenty features were selected as predictors of measles immunization defaulting in Uganda and Kenya. Phi-k correlation coefficient and significance were used because they provide a unified mathematically consistent metric capable of capturing both linear and nonlinear dependencies across categorical, ordinal, and interval variables without requiring separate statistical tests or manual encoding [38].

To reduce noise, the study used a significance matrix of each feature, multicollinear “twin” resolution, and phi-k correlation coefficient with the binary target (defaulting-H9) as criteria. For example, if 2 features had Q_k_>0.85 (Figure 3), it was assumed to be redundant, leading to dropping one Q_k_<0.05 or if or near zero towards the target variable, it was flagged as noise. Similarly, the significance threshold of z score<3.0 was used to discard statistically irrelevant predictors, hence retaining 11 features (Table 1).

**Figure 3.**
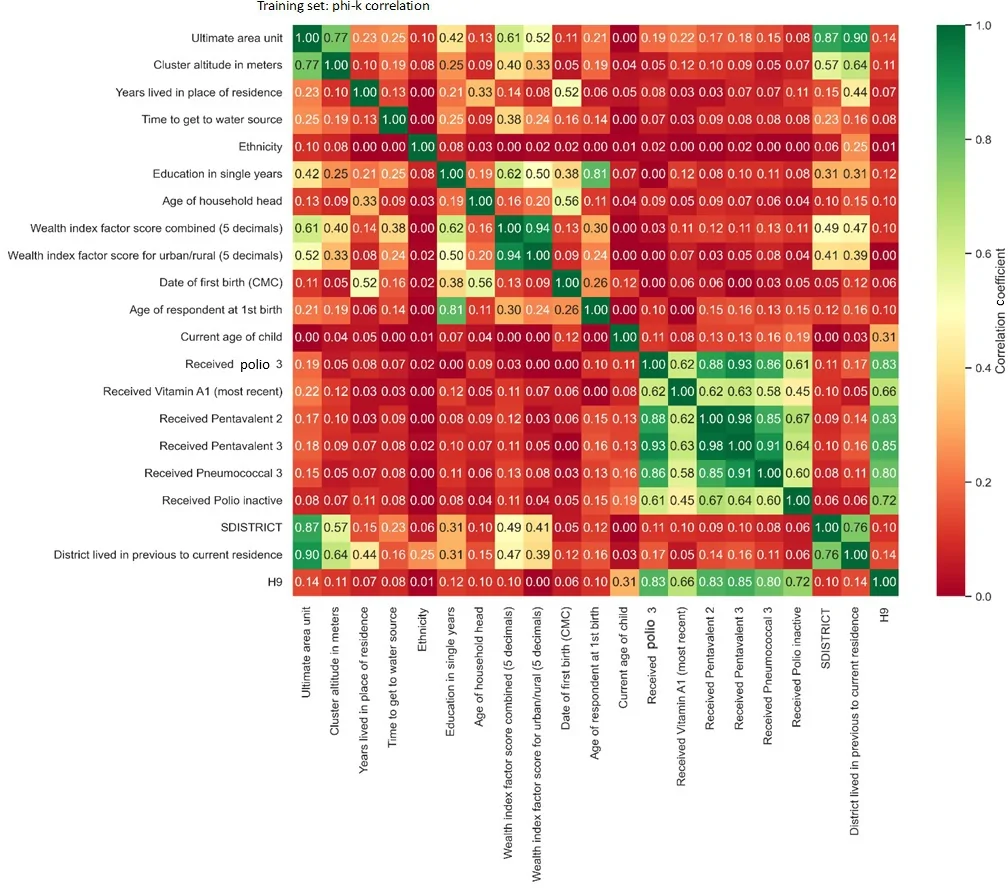
Phi-k correlation coefficient heap map. SDISTRICT: Sample district.

**Table 1.** Measles vaccination predictors and their status.

Feature	Target significance z-score	Global Phi-K	Status
Ultimate area unit	5.69	0.998	Drop (twin of sample district)
Cluster altitude in meters	3.99	0.993	Keep
Years lived in place of residence	1.77	0.992	Drop (not significant)
Time to get to water source	5.82	0.516	Keep
Ethnicity	0.66	0.984	Drop (not significant)
Education in single years	5.30	0.705	Keep
Age of household head	2.56	0.626	Drop (not significant)
Wealth index factor score combined	3.83	0.996	Keep
Wealth index factor score for urban/rural	0.55	0.631	Drop (not significant)
Date of first birth (CMC^a^)	1.54	0.987	Drop (not significant)
Age of respondent at 1st birth	4.76	0.840	Keep
Current age of child	23.52	0.446	Keep
Received Polio 3	64.34	0.995	Drop (twin of received Pentavalent 2)
Received vitamin A1 (most recent)	44.47	0.991	Keep
Received Pentavalent 2	67.49	0.997	Drop (twin of received Pentavalent 3)
Received Pentavalent 3	68.46	0.997	Keep
Received Pneumococcal 3	57.73	0.871	Drop (twin of Received Polio 3)
Received Polio inactive	49.16	0.657	Keep
Sample district	7.87	0.663	Keep
District lived in previous to current residence	6.44	0.997	Keep
H9	—^b^	—	Target

a CMC: century month code.

b Not applicable.

#### Data Discretization

The data discretization technique transformed numerical data into categories for measles defaulters and vaccinated. That is, vaccination date on card, reported by the mother, and vaccination marked on card were categorized as immunized (negative), while no or do not know were categorized as defaulters (positive).

#### Handling Vaccine Eligibility and Missing Values

Out of 8928 infants in the UDHS dataset, only 6793 infants were eligible for measles vaccination, while in Kenya 6403 were eligible. DHS has standardized placeholders (default value imputation) for categorizing missing values. This was adopted by this study for both UDHS and KDHS to ensure structural consistency and preserve critical behavioral context that traditional statistical imputation, like mean or mode, may inherently destroy.

#### Data Normalization

Normalization, also called feature scaling, is an important step that gives superior model performance for datasets with varying scales [39]. Models benefit from rescaling all attributes to have the same scale [40]. It standardizes the feature values to mitigate model bias due to scale variations [41] and optimizes algorithms [40]. The study used RobustScaler to transform the data, Xscaled=(x-median)/IQR where x is the original value. RobustScaler uses IQR to preserve the original data variance by ignoring the distracting effects of extreme values and is therefore not affected by outliers, unlike min-max [39].

#### Data Distribution and Balancing

The distribution of measles defaulters was 26.3% (1685/6403) and 22.1% (1504/6793) for Kenya and Uganda, respectively. The datasets had class imbalance; therefore, the SMOTE nominal continuous (SMOTE-NC) was used to balance the training sets after the split of training from test sets to avoid data leakage.

#### Rationale for Base Models’ Selection

This study considered diversity in base model selection that would capture different patterns in the data to handle complex and nonlinear relationships [20]. Deep neural network models like TabNet and MLP were used. MLP is a general-purpose black box neural network model known to be good for structured data. TabNet is an interpretable model with a sequential and attention-based feature selection at every step. This interpretability is key in health-related research like this study. Similarly, tree-based ensemble methods like AdaBoost and RFs were selected due to their capacity to combine multiple weaker learners sequentially to create a robust and high-performing model [29,42]. Lastly, logistic regression (LR) is a simple yet powerful linear model that provides a clear, interpretable relationship between features, while SVM is a kernel-based model that works by finding the optimal hyperplane [43].

#### Model Training

Experiments were done after dataset balancing and with cross-validation. The stratified k-fold cross-validation (CV), that is, k=5 fold, was used since CV offers better performance and less biased estimates [44]. K-fold CV results are reiterated with the mean score of the values of the model [44].

#### Model Classification and Threshold Optimization

Base algorithms were first trained at a default at 0.5 threshold as baselines. Predictions were initially generated as continuous raw probability scores, representing the calculated risk of an infant defaulting on their measles vaccination. These continuous probabilities were mapped into hard binary classification (1=defaulter, 0=immunized). A data-driven optimization strategy was implemented rather than relying on standard default parameters. The decision boundary was optimized dynamically using precision_recall_curve to maximize the localized (*F*_1_-score) harmonic mean of precision and recall on the test set.

### Model Evaluation

Evaluation establishes the level of excellence achieved by a proposed model [45]. This study employed metrics, including: accuracy, recall, precision, area under the receiver operating characteristic curve (AUROC), and confusion matrix.


(1) $$Accuracy=\frac{TP+TN}{TP+FP+FN+TN}$$

(2) $$Recall=\frac{TP}{TP+FN}$$

(3) $$Precision=\frac{TP}{TP+FP}$$

(4) $$F1\;Score=2\times \frac{Precision\times Recall}{Precision+Recall}$$

Where TP is true positive, FP is false positive, TN is true negative, and FN is false negative.

#### Confusion Matrix

A confusion matrix is a two-dimensional table that is helpful in classification problems [43] to present counts of how often each category is classified or misclassified [40]. The confusion matrix was instrumental in this study, given the imbalanced distribution.

#### SHAP Analysis

SHAP is a game theory approach that explains the output of a model [21]. It calculates Shapley values to fairly distribute the “payout” among features towards the model’s prediction. It provides local and global interpretability, which is crucial for building trust, especially in health-related research like this study and debugging models. A high SHAP value indicates a high probability of defaulting the measles vaccine, while a low value indicates receiving.

#### Hyperparameter Tuning

The advanced ensemble models had different individual base models (Table 2) that were first trained and tuned separately.

**Table 2.** Summaries of hyperparameters for the models.

Hyperparameter	Description	GB^a^	AB^b^	RF^c^	LR^d^	SVM^e^	DT^f^	MLP^g^	TabNet^h^
n_estimators	Number of boosting rounds (trees)	[50, 100, 200]	[50, 100, 200]	[50, 100, 200]	—^i^	—	—	—	—
learning_rate	—	[0.01, 0.1, 0.2]	[0.01, 0.1, 0.2]	—	—	—	—	—	—
max_depth	Max tree depth, -1 means no limit	[3,4,5]	—	[10, 20, None]	—	—	[5, 10, 20, None]	—	—
Max_features	No. of features to split.	—	—	[“:sqrt,” “log2”]	—	—	—	—	—
Min_samples_leaf	Minimum samples in leaf node	—	—	[1, 2, 4]	—	—	[1, 5, 10]	—	—
C	Penalty strength for misclassification	—	—	—	[0.01, 0.1, 1.0, 10.0, 100.0]	[0.1, 1.0, 10.0]	—	—	—
penalty	Controls type of regularization	—	—	—	[“l1,” “l2”]	—	—	—	—
kernel	Function to transform data	—	—	—	—	[“linear,” “rbf”]	**—**	—	—
gamma	Influence range of support vectors	—	—	—	—	[“scale,” “auto”]	—	—	—
criterion	Function to measure split quality	—	—	—	—	—	[“gini,” “entropy”]]	—	—
Hidden_layer_sizes	Tuple representing number of neurons in each hidden layer.	—	—	—	—	—	—	[(100), (50, 50)], (100, 50, 25)]	—
activation	Activation function relu (rectified linear unit)	—	—	—	—	—	—	[“tanh,” “relu”]	—
alpha	L2 regularization term coefficient added to loss function to penalize large weights and avoid overfitting	—	—	—	—	—	—	[0.001, 0.0001]	—
solver	Adaptive moment estimation (adam) stochastic gradient-based optimizer	—	—	—	—	—	—	[“adam,” “sgd”]	—
n_d	Width of the decision prediction layer	—	—	—	—	—	—	—	randint(8, 32)
n_a	Width of the attention embedding layer	—	—	—	—	—	—	—	randint(8, 32)
n_steps	Number of decision steps	—	—	—	—	—	—	—	randint(3, 10)
gamma	Relaxation parameter for attention	—	—	—	—	—	—	—	[1.3, 1.5, 2.0]
Lambda_sparse	Sparsity regularization	—	—	—	—	—	—	—	[0.001, 0.0001, 0.00001
Default value	—	[100, 0.1, 3]	[50, 1.0]	[500,20, 1, 1]	[1.0, l2]	[1.0, rbf, scale]	[None, 1, gini]	[100, “relu,” “adam,” 0.0001]	[8,8,3,1.3,0.001]

a GB: gradient boosting.

b AB: adaptive boosting.

c RF: random forest.

d LR: logistic regression.

e SVM: support vector machine.

f DT: decision tree.

g MLP: multilayer perceptron.

h TabNet: Tabular Network.

i Not applicable.

### Ethical Considerations

The DHS program granted the use of UDHS and KDHS datasets in accordance with the Institutional Review Board (IRB) approved procedures for DHS public use datasets (see ethical approvals in Multimedia Appendix 1). Human participant consent is not applicable in this study since secondary datasets were used (see ethical approvals in Multimedia Appendix 1).

## Results

### Overview

Models experimented with TabNet, RF, MLP, LR, GB, decision trees (DT), Support vector machine (SVM), and AB among others.

In Table 3, the SD for all metrics was very low, with the highest (0.0246) being in the acceptable range. This revealed a low variability and consistent performance across the five folds; therefore, models may generalize well to unseen data. GB was the best performing model with an *F*_1_-score of 0.7034 for Kenya, closely followed by SVM (*F*_1_-score=0.7013). For Uganda, RF outperformed others (*F*_1_-score=0.6520), closely followed by GB (*F*_1_-score=0.6495). Similarly, GB and RF had the highest accuracy, precision, and AUROC for the respective countries.

**Table 3.** Experimental results for individual models with k=5.

Model and country	Accuracy, mean (SD)	Precision, mean (SD)	Recall, mean (SD)	*F*_1_-score, mean (SD)	AUROC^a^, mean (SD)
LR^b^					
Kenya	0.8074 (0.0060)	0.6060 (0.0087)	0.7668 (0.0179)	0.6769 (0.0114)	0.8393 (0.0098)
Uganda	0.7534 (0.0058)	0.4611 (0.0096)	0.6748 (0.0209)	0.5478 (0.0133)	0.8156 (0.0068)
DT^c^					
Kenya	0.7528 (0.0057)	0.5257 (0.0099)	0.6309 (0.0235)	0.5731 (0.0056)	0.7136 (0.0049)
Uganda	0.7730 (0.0112)	0.4901 (0.0202)	0.6270 (0.0289)	0.5501 (0.0234)	0.7207 (0.0172)
RF^d^					
Kenya	0.8231 (0.0051)	0.6435 (0.0061)	0.7341 (0.0220)	0.6857 (0.0125)	0.8634 (0.0058)
Uganda	0.8356 (0.0073)	0.6132 (0.0136)	0.6961 (0.0268)	0.6520 (0.0184)	0.8706 (0.0098)
GB^e^					
Kenya	0.8284 (0.0086)	0.6448 (0.0124)	0.7739 (0.0243)	0.7034 (0.0168)	0.8715 (0.0063)
Uganda	0.8310 (0.0070)	0.6005 (0.0139)	0.7074 (0.0221)	0.6495 (0.0156)	0.8741 (0.0101)
AB^f^					
Kenya	0.8262 (0.0072)	0.6450 (0.0106)	0.7549 (0.0222)	0.6955 (0.0146)	0.8660 (0.0065)
Uganda	0.8176 (0.0079)	0.5688 (0.0154)	0.7320 (0.0212)	0.6399 (0.0134)	0.8668 (0.0093)
KNN^g^					
Kenya	0.8101 (0.0079)	0.6265 (0.0150)	0.6902 (0.0332)	0.6564 (0.0177)	0.8227 (0.0134)
Uganda	0.7879 (0.0043)	0.5169 (0.0076)	0.6383 (0.0166)	0.5712 (0.0105)	0.8089 (0.0070)
NB^h^					
Kenya	0.7913 (0.0093)	0.5880 (0.0152)	0.6926 (0.0202)	0.6359 (0.0164)	0.8216 (0.0103)
Uganda	0.5682 (0.0072)	0.3181 (0.0046)	0.8304 (0.0122)	0.4600 (0.0058)	0.7601 (0.0060)
SVM^i^					
Kenya	0.8288 (0.0081)	0.6483 (0.0117)	0.7638 (0.0220)	0.7013 (0.0158)	0.8495 (0.0102)
Uganda	0.8175 (0.0038)	0.5721 (0.0069)	0.6968 (0.0149)	0.6283 (0.0088)	0.8381 (0.0072)
MLP^j^					
Kenya	0.8188 (0.0057)	0.6277 (0.0090)	0.7662 (0.0246)	0.6899 (0.0122)	0.8652 (0.0083)
Uganda	0.8201 (0.0064)	0.5770 (0.0141)	0.7061 (0.0240)	0.6347 (0.0116)	0.8647 (0.0064)
TabNet^k^					
Kenya	0.8113 (0.0073)	0.6164 (0.0108)	0.7496 (0.0275)	0.6763 (0.0149)	0.8528 (0.0094)
Uganda	0.8114 (0.0091)	0.5573 (0.0175)	0.7294 (0.0283)	0.6313 (0.0136)	0.8628 (0.0095)

a AUROC: area under the receiver operating characteristic curve.

b LR: logistic regression.

c DT: decision tree.

d RF: random forest.

e GB: gradient boosting.

f AB: adaptive boosting.

g KNN: k-nearest neighbors

h NB: Naive Bayes

i SVM: support vector machine.

j MLP: multilayer perceptron.

k TabNet: Tabular Network.

### Confusion Matrix in the Context of This Study

When comparing models, this study’s primary focus was to achieve a high TP (predict the highest number of defaulters), minimal FN, which represents the zero dose of the vaccination schedule, potential dropouts that act as reservoirs for VPD, and minimizing false alarms (see Table 4).

Table 5 illustrates that GB outperformed other models by predicting 79% (267/337) of all defaulters and 18% (169/944) false alarms for Kenya. For Uganda, NB had the highest predicted defaulters at 78% (236/301); however, it had a high 47% (493/1058) false alarms. On the other hand, AB at 71% (215/301) TP and 14% (150/1058) false alarms closely followed TabNet and GB. Figure 4 and Table 6 present the Uganda and Kenya hyperparameter tuning results.

**Table 4.** Confusion matrix in the study context.

	Predicted negative (immunized)	Predicted positive (defaulter)
Actual negative (immunized)	True negative: correctly cleared	False positive: false alarm
Actual positive (defaulter)	False negative: missed defaulter	True positive: identified defaulter

**Table 5.** Experimental confusion matrix for individual models.

	RF^a^	MLP^b^	TabNet^c^	AB^d^	GB^e^	SVM^f^	NB^g^	KNN^h^	LR^i^	DT^j^
Kenya (total instances: 1281)										
True positive	253	261	262	258	267	265	235	247	258	226
False negatives	84	76	75	79	70	72	102	90	79	111
True negative	780	778	772	787	775	787	777	784	762	743
False positives	164	166	172	157	169	157	167	160	182	196
Uganda (total instances: 1359)										
True positive	205	199	211	215	208	202	236	186	196	189
False negatives	96	102	90	86	93	99	65	115	105	112
True negative	942	926	901	908	929	931	565	897	833	870
False positives	116	132	157	150	129	127	493	161	225	188

a RF: random forest.

b MLP: multilayer perceptron.

c TabNet: Tabular Network.

d AB: adaptive boosting.

e GB: gradient boosting.

f SVM: support vector machine.

g NB: naïve Bayes.

h KNN: k-nearest neighbor.

i LR: logistic regression.

j DT: decision tree.

**Figure 4.**
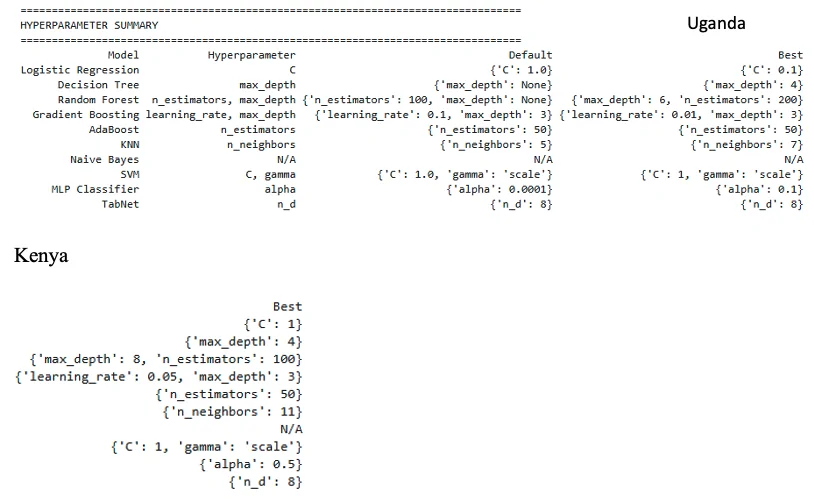
Hyperparameter summary for Uganda and Kenya.

**Table 6.** Grid search hyperparameter tuning results.

Model and country	Accuracy	Precision	Recall	AUC^a^	*F*_1_-score tuned	*F*_1_-score default parameters	Improvement (*F*_1_-score)
LR^b^							
Kenya	0.7955	0.5793	0.8131	0.8551	0.6765	0.6769	–0.0004
Uganda	0.7432	0.4514	0.7409	0.8189	0.5610	0.5478	0.0132
DT^c^							
Kenya	0.8298	0.6621	0.7211	0.8608	0.6903	0.5731	0.1171
Uganda	0.8440	0.6478	0.6478	0.8394	0.6478	0.5501	0.0977
RF^d^							
Kenya	0.8337	0.6615	0.7537	0.8789	0.7046	0.6857	0.0189
Uganda	0.8469	0.6431	0.7944	0.8851	0.6677	0.6520	0.0157
GB^e^							
Kenya	0.8345	0.6632	0.7537	0.8831	0.7056	0.7034	0.0022
Uganda	0.8418	0.6229	0.7243	0.8833	0.6697	0.6495	0.0202
AB^f^							
Kenya	0.8197	0.6183	0.8220	0.8829	0.7057	0.6955	0.0102
Uganda	0.8175	0.5626	0.7907	0.8718	0.6575	0.6399	0.0176
KNN^g^							
Kenya	0.8025	0.6105	0.6884	0.8407	0.6471	0.6564	–0.0093
Uganda	0.8219	0.6081	0.5515	0.8149	0.5784	0.5712	0.0072
NB^h^							
Kenya	0.7994	0.5905	0.7745	0.8379	0.6701	0.6359	0.0342
Uganda	0.7057	0.4016	0.6711	0.7608	0.5025	0.4600	0.0425
SVM^i^							
Kenya	0.8142	0.6055	0.8427	0.8753	0.7047	0.7013	0.0034
Uganda	0.8352	0.6222	0.6512	0.8404	0.6364	0.6283	0.0081
MLP^j^							
Kenya	0.8072	0.5926	0.8546	0.8750	0.6999	0.6899	0.010
Uganda	0.8256	0.5884	0.7076	0.8564	0.6425	0.6347	0.0078
TabNet^k^							
Kenya	0.8337	0.6649	0.7418	0.8704	0.7013	0.6763	0.0025
Uganda	0.8366	0.6215	0.6711	0.8632	0.6454	0.6313	0.0141

a AUC: area under the curve.

b LR: logistic regression.

c DT: decision tree.

d RF: random forest.

e GB: gradient boosting.

f AB: adaptive boosting.

g KNN: k-nearest neighbor

h NB: naive Bayes

i SVM: support vector machine.

j MLP: multilayer perceptron.

k TabNet: Tabular Network.

An improvement in model performance is observed in 7/9 models across all metrics after hyperparameter tuning. For example, TabNet had an improved *F*_1_-score of 2.5% (Kenya) and 1.41% (Uganda), RF at 1.89% (Kenya) and 1.57% (Uganda); DT at 11.72% (Kenya) and 9.77% (Uganda), among others, while only KNN and LR for Kenya performed best at default parameters. However, we noticed the reduced FN (defaulters; Tables 7 and 8); there was a high number of FP (false alarms) that necessitated further model refinement.

**Table 7.** Confusion matrix for the tuned models: Uganda (N=1359).

	LR^a^	DT^b^	RF^c^	AB^d^	GB^e^	KNN^f^	NB^g^	SVM^h^	MLP^i^	TabNet^j^
True positive	223	195	209	238	218	166	202	196	213	202
False Negatives	78	106	92	63	83	135	99	105	88	99
True Negative	787	952	942	873	926	951	757	939	909	935
False Positives	271	106	116	185	132	107	301	119	149	123

a LR: logistic regression.

b DT: decision tree.

c RF: random forest.

d AB: adaptive boosting.

e GB: gradient boosting.

f KNN: k-nearest neighbor.

g NB: naive Bayes.

h SVM: support vector machine.

i MLP: multilayer perceptron.

j TabNet: Tabular Network.

**Table 8.** Confusion matrix for the tuned models: Kenya (N=1281).

	LR^a^	DT^b^	RF^c^	AB^d^	GB^e^	KNN^f^	NB^g^	SVM^h^	MLP^i^	TabNet^j^
True Positive	274	243	254	277	254	232	261	284	288	250
False Negatives	63	94	83	60	83	105	76	53	49	87
True Negative	745	820	814	773	815	796	763	759	746	818
False Positives	199	124	130	171	129	148	181	185	198	126

a LR: logistic regression.

b DT: decision tree.

c RF: random forest.

d AB: adaptive boosting.

e GB: gradient boosting.

f KNN: k-nearest neighbor.

g NB: naive Bayes.

h SVM: support vector machine.

i MLP: multilayer perceptron.

j TabNet: Tabular Network.

Table 9 revealed that the advanced ensemble (custom soft voting and TabNet stacking) improved performance compared to the best-tuned base models. TabNet+ GB+RF→Soft voting outperformed all stacked ensembles with an area under the curve (AUC) of 0.8847 and *F*_1_-score of 0.7111 at an optimal threshold of 0.5125 for Kenya, while GB + RF+AB→TabNet was the best for Uganda with an AUC of 0.8867 and an *F*_1_-score of 0.6756 at an optimal threshold of 0.5163. Individual models seemed to have a higher accuracy; however, the confusion matrix revealed that the models were failing to identify measles vaccine defaulters (minority) but overwhelmingly predicting the vaccinated (majority class) despite the application of SMOTE-NC in training.

**Table 9.** Advanced ensemble learning model performance comparison.

Ensemble number	Base learner	Meta learner	Country	Accuracy	Precision	Recall	AUC^a^	*F*_1_-score
—^b^	AB^c^ (Best base)	—	Kenya	0.8197	0.6183	0.8220	0.8829	0.7057
—	GB^d^ (Best base)	—	Uganda	0.8418	0.6229	0.7243	0.8833	0.6697
1	TabNet^e^ +MLP^f^	LR^g^	Kenya Uganda	0.8009 0.8447	0.5823 0.6562	0.8605 0.6279	0.8787 0.8580	0.6946 0.6418
2	GB +RF^h^+AB	TabNet	Kenya Uganda	0.8189 0.8403	0.6224 0.6141	0.7923 0.7508	0.8730 0.8862	0.6971 0.6756
3	GB +RF+AB	LR	Kenya Uganda	0.8314 0.8359	0.6547 0.6021	0.7596 0.7641	0.8828 0.8881	0.7033 0.6735
4	TabNet +RF	LR	Kenya Uganda	0.8228 0.8366	0.6341 0.6070	0.7715 0.7442	0.8786 0.8864	0.6961 0.6687
5	TabNet +GB+RF	Soft voting	Kenya Uganda	0.8376 0.8418	0.6684 0.6280	0.7596 0.7010	0.8847 0.8847	0.7111 0.6625

a AUC: area under the curve.

b Not applicable.

c AB: adaptive boosting.

d GB: gradient boosting.

e TabNet: Tabular Network.

f MLP: multilayer perceptron.

g LR: logistic regression.

h RF: random forest.

Table 10 revealed that the proposed advanced ensembles GB + RF+AB→TabNet and TabNet + GB+RF→Soft voting with the highest *F*_1_-score correctly classified 3% for Uganda (8/301) and 1% for Kenya (2/337) more of the total TP that were previously misclassified by the best optimized base models, leading to a subtle improvement yet significant reduction in Type II errors (FNs). TabNet + GB+RF→Soft voting in Kenya presented the highest TN and the lowest TP. For Uganda, GB + RF+AB→TabNet had moderate FPs (false alarms). This was a trade-off since FPs are less harmful compared to failure to identify a TP. In the context of this public health study, it is the defaulters, TP and FN (unimmunized) that pose the risk of VPD outbreaks.

**Table 10.** Confusion matrix for advanced ensemble models.

	TabNet^a^+ MLP^b^→LR^c^	GB^d^ + RF^e^+AB^f^→TabNet	GB + RF+AB→LR	TabNet + RF→LR	TabNet + GB+RF→Custom soft voting
Kenya (1281 instances)					
True Positive	290	267	256	260	256
False Negatives	47	70	81	77	81
True Negative	736	782	809	794	817
False Positives	208	162	135	150	127
Uganda (1359 instances)					
True Positive	189	226	230	224	211
False Negatives	112	75	71	77	90
True Negative	959	916	906	913	933
False Positives	99	142	152	145	125

a TabNet: Tabular Network.

b MLP: multilayer perceptron.

c LR: logistic regression.

d GB: gradient boosting.

e RF: random forest.

f AB: adaptive boosting.

Figures 5 and 6 and Table 11 indicate that the current age of the child, vitamin A1, respondent’s education, time to water source, district lived previously, and current district were the most influential global features in Kenya, respectively. In Uganda, vitamin A1, current age of the child, Polio inactive, Pentavalent 3, current district, and respondent’s education were the most influential factors to defaulting, respectively.

The statistical validation performance comparison based on McNemar test between Kenya and Uganda in Table 12 revealed that, in Kenya, a significant marginal performance was gained by TabNet + GB+RF ->Soft Voting over the tuned best base (AB; Δ*F*_1_-score=0.0054; *P*=.007; *χ*^2^_1_=7.224; Cohen g [effect size]=0.1716; CI for Δ *F*_1_-score spans zero −0.0156 to 0.0260). The absolute magnitude of improvement remains tight yet nonrandom pattern of corrected errors confirms the advanced ensemble’s structural advantage in Kenya. Conversely, in Uganda, the TabNet hybrid demonstrated that the algorithmic complexity does not universally guarantee statistical superiority. While GB + RF+AB→TabNet exhibited a slightly higher nominal improvement of Δ *F*_1_-score=0.0059, the 95% bootstrap CI again captures zero (−0.0113 to 0.0222). Crucially, the McNemar test registered no statistical significance (*χ*^2^_1_=0.033; *P*=.86) with weak effect size (Cohen g) of 0.0333. It reveals that the differences in classification error between the best tuned base model (GB) and the TabNet hybrid may be stochastic. This also indicates that the additional computational overhead of the advanced ensemble architecture fails to provide a meaningful predictive advantage in this specific context.

**Figure 5.**
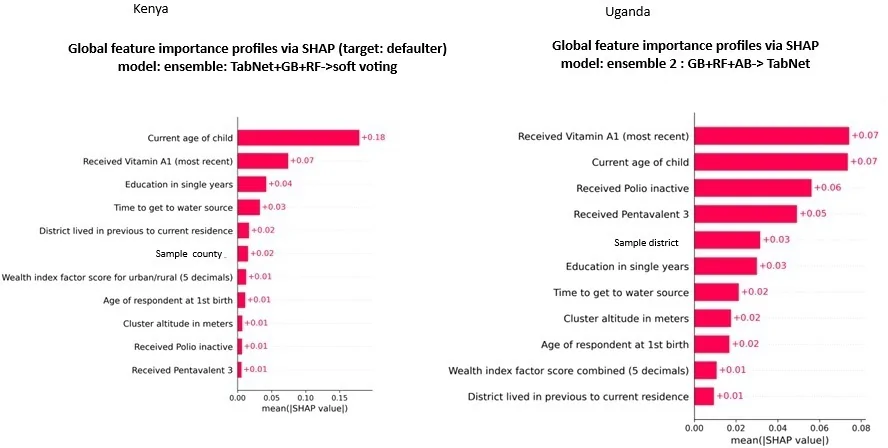
Global feature importance. SHAP: Shapley additive explanations.

**Figure 6.**
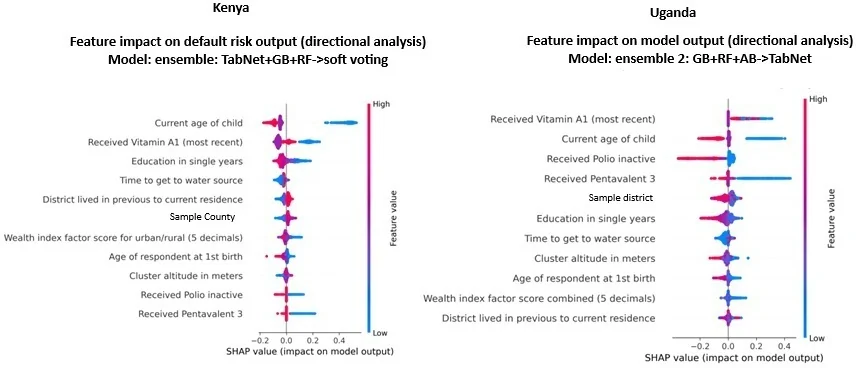
Feature impact on model output. SHAP: Shapley additive explanations.

**Table 11.** Ranked feature importance.

Country and feature	Mean SHAP^a^ value
Kenya	
Current age of child	0.179472
Received vitamin A1 (most recent)	0.074740
Education in single years	0.042691
Time to get to water source	0.033265
District lived in previous to current residence	0.017289
Sample county	0.015859
Wealth index factor score for urban/rural	0.013037
Age of respondent at 1st birth	0.011565
Cluster altitude in meters	0.007374
Received polio inactive	0.006813
Received Pentavalent 3	0.006167
Uganda	
Received vitamin A1 (most recent)	0.074347
Current age of child	0.073706
Received polio inactive	0.056320
Received Pentavalent 3	0.049364
Sample district	0.031718
Education in single years	0.030147
Time to get to water source	0.021435
Cluster altitude in meters	0.017715
Age of respondent at 1st birth	0.016960
Wealth index factor score for urban/rural	0.010812
District lived in previous to current residence	0.009542

a SHAP: Shapley additive explanations.

**Table 12.** Statistical validation comparison.

Country	Model	delta *F*_1_- score (95% CI)	Cohen g (effect size)	McNemar chi-square	*P* value	Significance
Kenya	Tuned base (AdaBoost) vs best (ensemble: TabNet^a^+GB^b^+RF^c^ ->soft voting)	0.0054 (-0.0156 to 0.0260)	0.1716	7.224 (1)	.007	Significant
Uganda	Tuned Base (GB) versus best (ensemble: GB+RF+AB^d^ ->TabNet)	0.0059 (-0.0113 to 0.0222)	0.0333	0.033 (1)	.86	Not significant

a TabNet: Tabular Network.

b GB: gradient boosting.

c RF: random forest.

d AB: adaptive boosting.

## Discussion

### Principal Findings

Experimental results for individual standard models revealed that GB was the best with an *F*_1_-score of 0.7034, closely followed by SVM at 0.7013, AB at 0.6955 for Kenya. For Uganda, RF outperformed others at 0.6520, closely followed by GB at 0.6495. Similarly, GB and RF had the highest accuracy, precision, and AUROC for the respective countries. These results resonate with [19,34,46], who indicated that the tree-based ensembles like RF were the best performing models. In addition, just like [47], tree-based models outperformed deep learning methods. NB was worst at 0.4600 (Uganda) and DT 0.5731 (Kenya).

Similarly, an improved performance among individual models was realized after hyperparameter tuning at a customized threshold. For example, TabNet had an improved (see Table 6) *F*_1_-score of 0.025 (Kenya) and 0.0141 (Uganda); RF at 0.0189 (Kenya) and 0.0157 (Uganda); DT at 0.1172 (Kenya) and 0.0977 (Uganda), among others. On the contrary, KNN and LR for Kenya performed best at standard parameters. Despite the improvement, however, the confusion matrices (Tables 8 and 9) revealed a high number of FP (false alarms) as a trade-off for reduced FN (defaulters), which necessitated further model refinement.

The proposed TabNet + GB+RF→soft voting for Kenya and GB + RF+AB→TabNet for Uganda achieved the highest AUC of 0.8847, *F*_1_-score of 0.7111 and AUC of 0.8867, *F*_1_-score of 0.6756 compared to the best optimized individual models AB AUC of 0.8829, *F*_1_-score of 0.7057 (Kenya) and GB AUC of 0.8829, *F*_1_-score of 0.6697 (Uganda) respectively. Similarly, the confusion matrix revealed that GB + RF+AB→TabNet and TabNet + GB+RF→soft voting correctly classified more TP compared to the best tuned models, that is; 8 of 301 (3%) infants in Uganda and 2 of 337 (1%) in Kenya. Our results resonate with [47], whose study of predicting Pentavalent3 dropout in East Africa revealed that the all-stacked ensemble models outperformed individual learners benchmarking RF, generalized linear model (GLM), and Extreme Gradient Boosting (XGBoost) as meta learners [47].

In Kenya, the advanced ensemble model was statistically significant and supported by a moderate Cohen g effect of 0.1716. Conversely, the performance in Uganda was stochastic indicating that the ensemble architecture provided no statistically valid predictive advantage in that context.

### Clinical Justification for Threshold Modification

Given the class imbalance in the datasets, the reliance on the default 0.5 threshold classification to determine the binary outcome of defaulter and immunized, assuming a perfectly symmetrical class distribution, would present a vulnerability. Similarly, it assumes an equivalent real-world cost for type I (FP) and type II (FN) errors, which is not the case in this study. A 0.5 threshold normally produces a high but deceptive accuracy biased towards the majority class while failing to identify the minority. To counteract this bias, we implemented the automated “find best threshold,” shifting the decision boundary away from an arbitrary midpoint and aligning it to the mathematical maximum *F*_1_-score.

This pipeline adaptively raised the threshold above the standard 0.5 (TabNet + RF+GB→soft voting at 0.5125 and GB + RF+AB→TabNet at 0.5163), indicating models’ ability to separate the two classes well (see Figure 7). The models are neither completely drowning in the majority class nor suffering from massive probability suppression. It also indicates the models confidently push TP cases past the 0.5 mark without accidentally dragging a flood of FPs along. This trade-off was necessary since FNs are clinically more costly. FN results mean either a zero dose or a defaulter reduces herd immunity. Conversely, a FP is negligible since the child is already immunized, posing no VPD outbreak to the community.

**Figure 7.**
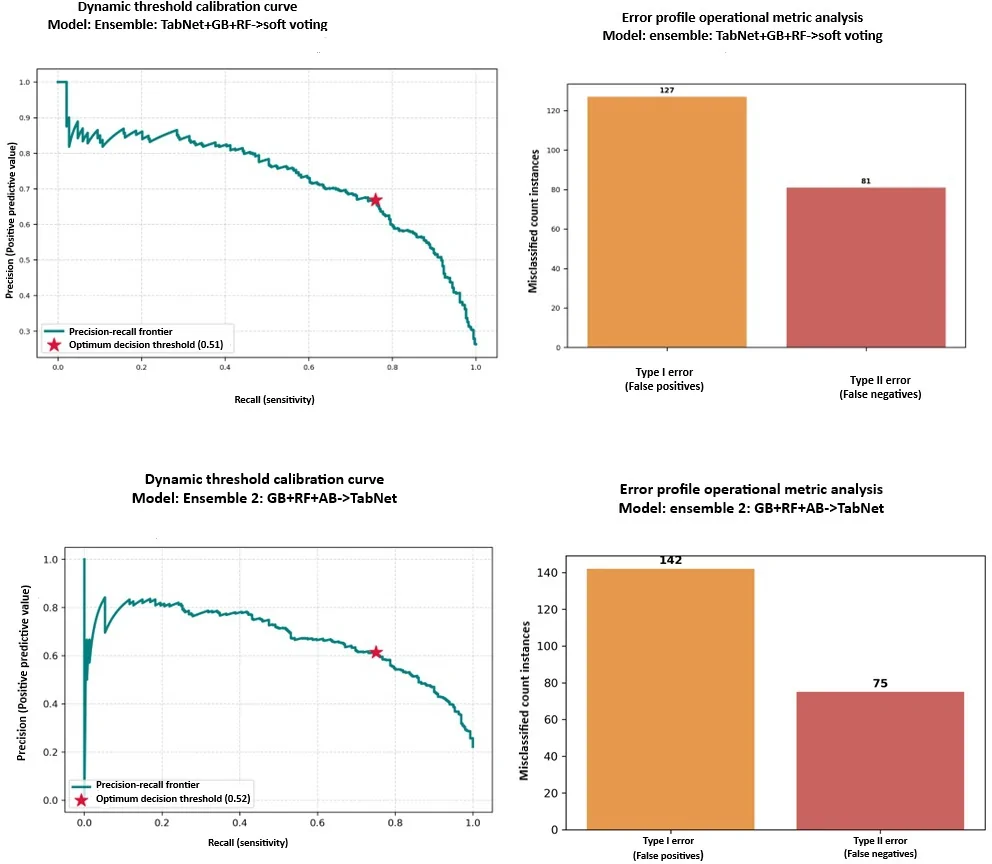
Best advanced ensemble dynamic threshold calibration and error profiling.

### Model Interpretability

The current study, like the one by Alemayehu et al [47], used SHAP to establish the global feature importance benchmarking the best advanced ensemble models. Experimental results using TabNet + GB+RF→Custom soft voting ensemble indicated current age of a child, most recent vitamin A1, education, time to water source, and district lived previously as the most influential global predictors in Kenya, with a mean absolute SHAP value (MASV) of 0.1795, 0.0747, 0.0427, 0.0333 and 0.0173, respectively. For Uganda, GB + RF+AB→TabNet revealed vitamin A1, age of child, polio inactive, Pentavalent3, and district were the most influential predictors at MASV of 0.0743, 0.0737, 0.0563, 0.0494, and 0.0317, respectively. Generally, the current age of a child and the most recent vitamin A1 administration are key universal predictors, closely followed by the district and the respondent’s education. The respondent’s education often correlates with the ability to understand the significance of adherence to the immunization schedule. On the other hand, as the child gets older, the chances of getting immunized increase, as a previously missed opportunity due to vaccine stockout or parents’ unavailability can be corrected at the following visit.

However, the influence of other features diverges between the two countries, reflecting unique socioeconomic and structural differences. For example, Polio inactive was a key predictor in Uganda and least in Kenya, and similarly, district lived previously was influential in Kenya and least in Uganda. This result reverberates with the study by Alemayehu et al [47], who stated that feature influence is context-dependent and advised that immunization drop-out policies and interventions should be tailored to subgroups or countries.

The proposed advanced ensembles present the opportunity to reduce the zero dose and immunization schedule defaulting since they could accurately predict 76% for Kenya (256/337) and for Uganda (226/301) of the potential defaulters while minimizing the false alarms. They also presented the highest *F*_1_-score, balancing precision and recall. FPs (immunized predicted as unimmunized) may not cause an outbreak or threat, but can raise the budget of immunizing already vaccinated infants, causing a financial loss due to a false alarm. Therefore, models like the proposed advanced ensembles are ideal since they minimize both the FN and FP.

### Conclusion

The proposed advanced ensemble learning methods improved performance compared to the best individual base models. In addition, they present an opportunity to reduce the zero dose and scheduled immunization defaulting since they could accurately predict 256 out of 337 (76%) infants in Kenya and 226 out of 301 (75%) infants in Uganda of all the potential defaulters that act as reservoirs for measles and pose a risk of measles outbreaks.

For interpretability, SHAP indicated that the age of a child and the most recent vitamin A1 administration are universal key predictors in both countries. However, other factors varied drastically by country. These findings emphasize the need for context-specific interventions rather than a one-size-fits-all approach to measles defaulting.

### Recommendations

Based on the SHAP advanced ensemble explainability, the study recommends distinct and localized public health strategies in the 2 countries. In Kenya, the study recommends outreaches in areas with distant water sources, low literacy, and migrating families. Conversely, in Uganda, where immunization history was a predominant predictor, embedding proactive alerts to would-be defaulters and outreaches to high-default districts are recommended. Since the current age of a child and vitamin A1 were universally crucial drivers in both countries, timely reminders as infants reach their peak window of age vulnerability are ideal.

### Clinical Utility and Proposed Tool Integration With DHIS2-EPIVAC

One of the proposed stack ensemble GB +RF+AB→TabNet was used to develop a pilot prototype application that proactively flags infants at risk of defaulting basic RI in Uganda (Figure 8). This functional, decisive tool can be used to provide targeted support, like sending reminders to parents or guardians whose infant was predicted to have a likelihood of defaulting. In addition, health workers would proactively schedule outreaches according to the concentration of many likely defaulters using the analytics (see district vs immunization status illustration in Figure 8). This can enable health workers to optimize immunization program delivery to high-default areas that can prevent sporadic VPD outbreaks and related mortality. This tool can also be integrated with EPIVAC (expanded program on immunization vaccination e-registry), a digital vaccination registry in Uganda built on District Health Information Software 2 (DHIS2) that captures individual longitudinal records nationally, like immunization schedule and maternal details, among others. Its operationalization can support early warning and response, just like malaria, climate change, machine learning, and modeling on DHIS2. Figure 8 illustrates the TabNet stack ensemble model prototype.

**Figure 8.**
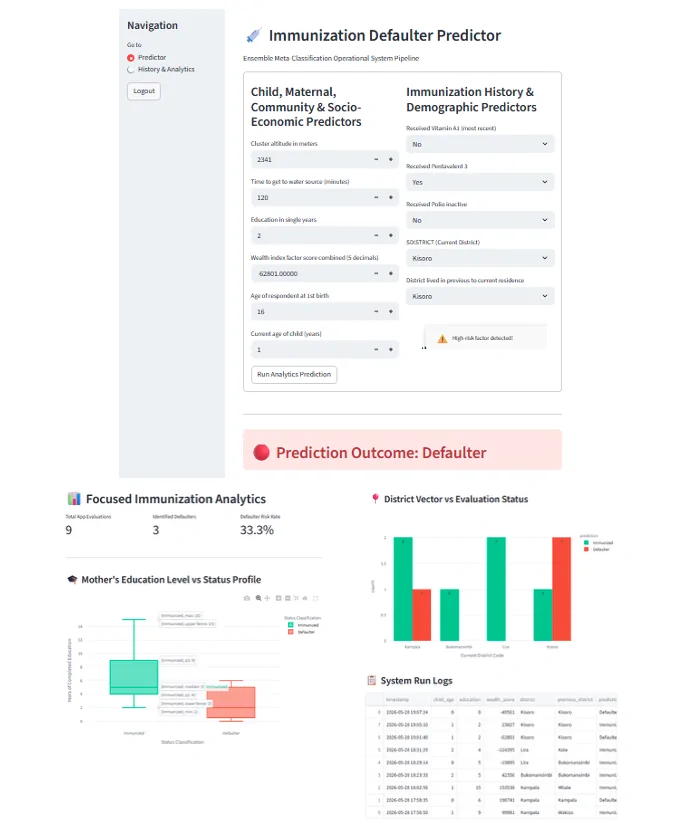
Screenshot of the basic routine immunization defaulter predictor tool and analytics of dummy records.

### Study Limitations

SHAP values represent the advanced ensemble model’s internal predictive logic that was based only on the used features, but the real-world measles defaulting may have other factors that may influence defaulting that this model may not have accounted for. Consequently, the high importance values may reflect training correlations rather than direct epidemiological drivers of defaulting.

## Supplementary material

10.2196/95727 Multimedia Appendix 1 Immunization schedule and feature correlation.

## Abbreviations

AB: adaptive boosting
AUC: area under the curve
AUROC: area under receiver operating characteristic curve
CV: cross-validation
DHIS2: District Health Information Software 2
DHS: Demographic and Health Survey
DT: decision trees
EA: enumeration area
EPIVAC: expanded program on immunization vaccination e-registry
FN: false negative
FP: false positive
GB: gradient boosting
GLM: generalized linear model
IRB: Institutional Review Board
KDHS: Kenya Demographic and Health Survey
KNBS: Kenya National Bureau of Statistics
LR: logistic regression
MASV: mean absolute SHAP value
MLP: multilayer perceptron
NPHC: National Population and Housing Census
RBA: resource-based approach
RCM: rapid coverage monitoring
RF: random forest
RI: routine immunization
SHAP: Shapley additive explanations
SMOTE: synthetic minority over-sampling technique
SMOTE-NC: synthetic minority over-sampling technique nominal continuous
SVM: support vector machine
SVR: support vector regression
TabNet: Tabular Network
TN: true negative
TP: true positive
UBOS: Uganda Bureau of Statistics
UDHS: Uganda Demographic and Health Survey
VPD: vaccine-preventable disease
XGBoost: Extreme Gradient Boosting
