# Supplementary material for: Predicting Measles Vaccination Defaulting in Uganda and Kenya: Comparative Evaluation of Custom Soft Voting and TabNet Stacked Ensemble Architectures
Source: JMIR AI. 2026 Oct 13;5:e95727. doi: 10.2196/95727 (PMC13660459; doi:10.2196/95727)
Supplement: Supplementary file 1 [file ai-v5-e95727-s001.docx]

**SUPPLEMENTARY INFORMATION**

| Visit | Age | Vaccine | Disease Prevented | How and where vaccination is administered |
| --- | --- | --- | --- | --- |
| 1st | At Birth | Polio 0 | Polio | 2 Drops in the mouth |
|  |  | BCG | Tuberculosis | Injection on the right (upper ram) |
| 2^nd^ | At 6 weeks  (1.5 months) | Pentavalent 1 | Diphtheria, Whooping cough, Tetanus, Hepatitis B, Haemophilus influenzae type B | Injection on the left thigh |
|  |  | Polio 1 | Polio | 2 Drops in the mouth |
|  |  | Pneomococcal conjugate vaccine 10 (PCV 1) | Meningitis and Pneumonia (caused by Streptococcal Pneumoniae) | Injection on the right thigh |
|  |  | Rotavirus Vaccine 1 | Diarrhoea (caused by Rotavirus | Slow release into the mouth (Baby sucks) |
| 3^rd^ | At 10 weeks (2.5 months) | Polio 2 | Polio | 2 Drops in the mouth |
|  |  | Pentavalent 2 | Diphtheria, Whooping cough, Tetanus, Hepatitis B, Haemophilus influenzae type B | Injection on the left thigh |
|  |  | Pneomococcal conjugate vaccine 10 (PCV 2) | Meningitis and Pneumonia (caused by Streptococcal Pneumoniae) | Injection on the right thigh |
|  |  | Rotavirus Vaccine 2 | Diarrhoea (caused by Rotavirus) | Slow release into the mouth (Baby sucks) |
| 4^th^ | At 14 weeks (3.5 months) | Polio 3 | Polio | 2 Drops in the mouth |
|  |  | Injectable Polio Vaccine (IPV) | Polio | Injection on the left thigh |
|  |  | Pentavalent 3 | Diphtheria, Whooping cough, Tetanus, Hepatitis B, Haemophilus influenzae type B | Injection on the left thigh |
|  | At 6 months and every 6 months until the child is 5 years old | Vitamin A Supplement | Prevent blindness and strengthen resistance against other diseases | Drops in the mouth |
| 5^th^ | At 9 months | Measles vaccine | Measles | Injection on the left arm |

Supplementary Table 1: Shows the routine immunization schedule for infants under one year

**ETHICAL APPROVALS**
